# Accuracy Assessment of Oura Ring Nocturnal Heart Rate and Heart Rate Variability in Comparison With Electrocardiography in Time and Frequency Domains: Comprehensive Analysis

**DOI:** 10.2196/27487

**Published:** 2022-01-18

**Authors:** Rui Cao, Iman Azimi, Fatemeh Sarhaddi, Hannakaisa Niela-Vilen, Anna Axelin, Pasi Liljeberg, Amir M Rahmani

**Affiliations:** 1 Department of Electrical Engineering and Computer Science University of California Irvine, CA United States; 2 Department of Computing University of Turku Turku Finland; 3 Department of Nursing Science University of Turku Turku Finland; 4 Department of Computer Science University of California Irvine, CA United States; 5 School of Nursing University of California Irvine, CA United States

**Keywords:** electrocardiography, ECG, wearable device, heart rate variability, Oura smart ring

## Abstract

**Background:**

Photoplethysmography is a noninvasive and low-cost method to remotely and continuously track vital signs. The Oura Ring is a compact photoplethysmography-based smart ring, which has recently drawn attention to remote health monitoring and wellness applications. The ring is used to acquire nocturnal heart rate (HR) and HR variability (HRV) parameters ubiquitously. However, these parameters are highly susceptible to motion artifacts and environmental noise. Therefore, a validity assessment of the parameters is required in everyday settings.

**Objective:**

This study aims to evaluate the accuracy of HR and time domain and frequency domain HRV parameters collected by the Oura Ring against a medical grade chest electrocardiogram monitor.

**Methods:**

We conducted overnight home-based monitoring using an Oura Ring and a Shimmer3 electrocardiogram device. The nocturnal HR and HRV parameters of 35 healthy individuals were collected and assessed. We evaluated the parameters within 2 tests, that is, values collected from 5-minute recordings (ie, short-term HRV analysis) and the average values per night sleep. A linear regression method, the Pearson correlation coefficient, and the Bland–Altman plot were used to compare the measurements of the 2 devices.

**Results:**

Our findings showed low mean biases of the HR and HRV parameters collected by the Oura Ring in both the 5-minute and average-per-night tests. In the 5-minute test, the error variances of the parameters were different. The parameters provided by the Oura Ring dashboard (ie, HR and root mean square of successive differences [RMSSD]) showed relatively low error variance compared with the HRV parameters extracted from the normal interbeat interval signals. The Pearson correlation coefficient tests (*P*<.001) indicated that HR, RMSSD, average of normal heart beat intervals (AVNN), and percentage of successive normal beat-to-beat intervals that differ by more than 50 ms (pNN50) had high positive correlations with the baseline values; SD of normal beat-to-beat intervals (SDNN) and high frequency (HF) had moderate positive correlations, and low frequency (LF) and LF:HF ratio had low positive correlations. The HR, RMSSD, AVNN, and pNN50 had narrow 95% CIs; however, SDNN, LF, HF, and LF:HF ratio had relatively wider 95% CIs. In contrast, the average-per-night test showed that the HR, RMSSD, SDNN, AVNN, pNN50, LF, and HF had high positive relationships (*P*<.001), and the LF:HF ratio had a moderate positive relationship (*P*<.001). The average-per-night test also indicated considerably lower error variances than the 5-minute test for the parameters.

**Conclusions:**

The Oura Ring could accurately measure nocturnal HR and RMSSD in both the 5-minute and average-per-night tests. It provided acceptable nocturnal AVNN, pNN50, HF, and SDNN accuracy in the average-per-night test but not in the 5-minute test. In contrast, the LF and LF:HF ratio of the ring had high error rates in both tests.

## Introduction

### Background

Wearable devices are widely used for continuous monitoring of health parameters, by which individuals’ health and well-being can be assessed [1,2]. Heart rate (HR) and HR variability (HRV) are essential parameters that can be collected noninvasively, indicating information about the cardiorespiratory and autonomic nervous systems. HRV is the variation in the time interval between adjacent heartbeats, also known as interbeat interval (IBI) [3]. Using the IBI, various parameters can be extracted, such as the root mean square of successive differences (RMSSD), SD of beat-to-beat intervals (SDNN), and the percentage of successive beat-to-beat intervals that differ by more than 50 ms (pNN50), each of which reveals various cardiovascular events and problems [4]. For example, HRV parameters have been shown to be predictors of mortality after myocardial infarction [5] and the mode of death in chronic heart failure [6]. Studies also indicated that HRV parameters are associated with diabetes [7], cardiovascular autonomic imbalance [8], and in pregnant women with pre-eclampsia [9], to mention a few. Moreover, HRV parameters are significantly correlated with sleep stage [10], sleep quality [11], and stress levels [12,13].

HR and HRV monitoring can be performed by leveraging noninvasive and low-cost methods. Electrocardiography (ECG) is a conventional method to record the heart’s electrical activities, using electrodes attached to the chest and limbs [14]. ECG is a gold standard method for collecting heartbeats and IBI, as the collected electrical signals can clearly indicate depolarization of the ventricular muscles (ie, R-peak). However, the ECG method cannot be used for long-term or remote health monitoring owing to the complicated setup as ECG electrodes need to be attached to the user’s limbs or chest all the time. Loose or misplaced electrode connections in the monitoring also negatively affect signal quality. Photoplethysmography (PPG) is another technique used to collect HR and HRV [15]. Different studies have focused on monitoring and extraction of PPG signals [16-18]. As an optical technique, PPG measures cyclical oscillations in the skin’s blood flow by emitting light to the skin and absorbing light reflection via a light detector [19]. The light emitter and detector can be placed on the user’s wrist or finger for data collection. PPG is easy to implement in remote health monitoring systems, and it is already available in various wearable devices in the market, such as smartwatches and rings.

Several clinical and commercial PPG-based smart wearable devices have been proposed in the past few years, enabling the monitoring of vital signs outside conventional clinical settings. Studies have exploited wearables such as Garmin, Fitbit, and Apple watches in clinical trials as well as in different population-based studies [20-22]. The use of wearable devices is expected to increase even further as they become smaller, lighter, and more energy-efficient with sufficient battery capacity and internal data storage. In particular, smart rings such as the Oura Ring [23] have recently drawn attention for use in remote health and physical activity monitoring, such as COVID-19 research at the University of California San Francisco [24] and players’ health data monitoring in the National Basketball Association and Women's National Basketball Association league [25].

Wearable devices require a high level of accuracy and reliability, particularly if they are used in health monitoring apps. However, these devices are susceptible to artifacts, resulting in poor data collection and subsequently invalid health parameters. This problem is further exacerbated by environmental noise and motion artifacts in PPG-based monitoring [19,26]. Unfortunately, such artifacts are inevitable in free-living conditions, as the user might engage in various physical activities in different environments.

The accuracy of different HRV parameters depends on multiple factors in the signals. For example, RMSSD shows short-term variations in the IBI signal, and the accuracy is affected if a small portion of the signal is distorted [4]. In contrast, SDNN indicates the long-term signal variation, so the outliers, affecting the variation of the signal, would negatively impact its accuracy. Moreover, frequency domain features are significant for assessing the cardiovascular and nervous systems, for example, low frequency (LF) and high frequency (HF) are indicators of stress states, hypertension, and Parkinson disease severity [27,28]. These features indicate the power of the IBI in specific frequency bands. Therefore, they are distorted if interference with the same frequency is added to the signal. Such different characteristics of HRV parameters necessitate the evaluation of HRV parameters separately. Consequently, a more extensive assessment is required to investigate HRV measurements in remote monitoring.

Various studies in the literature have investigated the validity of wristbands—such as Apple Watch, Huawei Watch, and Microsoft Band 2—in terms of the quality of PPG and HRV measurements [29-32]. However, the validation of smart rings, which use finger-based PPG, is limited. Mehrabadi et al [33] assessed the nonstaging sleep parameters collected by the Oura Ring in comparison with a medically approved actigraphy device. They showed that the sleep parameters of the ring were significantly correlated with those obtained from the Actigraph. Kinnunen et al [34,35] investigated the Oura Ring via overnight data collection. The study showed good agreement between the Oura Ring and the ECG monitoring device. However, the assessments were restricted to the nocturnal HR and RMSSD reported by the ring. Other parameters, such as the IBI or frequency domain HRV parameters, were not considered.

### Objectives

In this study, we have comprehensively assessed the validity of the Oura Ring in terms of HR and multiple HRV parameters during sleep. The ring was evaluated against a medical grade chest ECG monitor. The study, approved by the ethical committee, included overnight home-based monitoring of 35 healthy individuals from whom the HR and IBI values were collected. We extracted HR, RMSSD, average of all normal heart beat intervals (AVNN), SDNN, pNN50, LF, HF, and LF:HF ratio from the ring and ECG monitor. Then, we evaluated the parameters obtained from the 2 devices in a 5-minute test and an average-per-night test. The parameters were compared using a linear regression method, Pearson correlation coefficient, and Bland–Altman plot. Finally, we have discussed the obtained results, the validity of monitoring these parameters in everyday settings, and the limitations of the study. In summary, the main contributions of this study are as follows:

1. We investigated the validity of the Oura Ring in terms of nocturnal HR and multiple HRV, compared with a medical grade chest ECG monitor.
2. We conducted a 1-day study where 35 healthy individuals were monitored at home.
3. We analyzed the HR and HRV parameters in 5-minute and average-per-night tests using a linear regression method, Pearson correlation coefficient, and Bland–Altman plot.

## Methods

### Study Design

The assessment of HR and HRV measurements collected from the Oura Ring was performed in an observational study in free-living conditions with a convenience sample of healthy individuals. The measurements were evaluated in comparison with a gold standard ECG monitor. Recruitment took place during July and August 2019 in southwest Finland.

### Participants and Recruitment

A total of 46 healthy volunteer adults—including 23 women and 23 men—were recruited in this study. The exclusion criteria were as follows: (1) diagnosed cardiovascular disease, (2) symptoms of illness during the recruitment time, (3) restriction in physical activity, and (4) restriction on using wearable devices. The average age and BMI of the selected participants were 32.3 (SD 6.4) years and 24.9 (SD 4.5) kg/m^2^, respectively. In this setup, we focused on healthy people to evaluate the accuracy of the ring, as diseases (arrhythmias) alter the shape of the PPG signal [36] and affect the regular accuracy of the ring.

In face-to-face meetings, the participants were informed about the study’s detailed information, including the purpose of the study and use of wearable devices. The participants were asked to wear an Oura Ring and a Shimmer3 ECG monitor for 1 day. Measurements were conducted during normal life. A total of 11 participants were excluded from the data analysis owing to technical and practical issues, for example, the ECG electrodes were not adequately attached to the skin during sleep. Consequently, data from 35 participants (women: 19/35, 54%; men: 16/35, 46%) were included in the analysis.

### Data Collection

The home-based data collection was performed using 2 wearable devices, that is, the Oura Ring [23] and Shimmer device [37]. The participants were asked to wear 1 Oura Ring on 1 finger of the nondominant hand. The Shimmer unit was placed on the chest of each participant using a chest strap. A total of 4 electrodes were attached to collect 3 bipolar limb leads during the monitoring. More details of the setup can be found in the study by Burns et al [38]. Moreover, the participants were asked to complete a short background questionnaire before starting the monitoring. They were also asked to report events during the study, for example, if the devices were removed from the finger or chest. In addition to the verbal instructions, the participants received written guidelines for using the devices.

Oura Ring is a commercial wearable device, collecting PPG, acceleration, and body temperature data to measure HR, respiratory rate, HRV, sleep parameters, and intensity of physical activity. The ring is small (2.55 mm thickness), light (4-6 g), and easy to use for continuous monitoring [35]. Its battery can support 5-7 consecutive days of monitoring with one battery charge. The ring uses Bluetooth to send data to the Oura Android or iOS operating system mobile app and cloud server. The data can be accessed through the mobile app or the server. In this study, we extracted the data from the Oura cloud [35].

The Shimmer3 ECG is the baseline device selected in this study to evaluate the HR and HRV of the ring. The device is light (31 g) and has compact dimensions (65 mm×32 mm×12 mm) [37]. The Shimmer3 ECG unit can be configured to measure ECG, accelerometer, and gyroscope data continuously. The device has a sufficient battery life and internal memory to perform monitoring for an entire day. We selected 512 Hz as the sampling frequency for ECG data collection [39,40]. The sampling frequency is sufficient to accurately extract HR and HRV [41]. Data were extracted from the device after the monitoring [37].

### Data Analysis

#### Oura Ring

The Oura Ring provides various parameters regarding the health, physical activity, and sleep of the user. PPG-based wearable devices (including Oura) collect noise along with the signals of interest, particularly in home-based monitoring. In this study, we assessed the accuracy level or noise level of HR and HRV measurements. The ring provides HR and RMSSD when the user is sleeping, and the values are reported every 5 minutes. More details about the HR and RMSSD calculation can be found in the study by Shaffer et al [4]. The ring also provides an IBI signal [42]. We used the 5-minute window of the IBI signal to calculate the time domain parameters (ie, AVNN, SDNN, and pNN50) and frequency domain parameters (ie, LF, HF, and LF:HF ratio). The HRV parameters are presented in Table 1. It should be noted that the ring preprocessed the signals and provided confidence values, demonstrating the validity of the IBI signals. We calculated the HRV of the 5-minute IBI signals, if at least 30% of the signal is valid [35].

**Table 1:** Heart rate variability parameters.

Parameter	Units	Description
NN^a^ interval	ms	Time interval between 2 successive normal heartbeats
RMSSD^b^	ms	The RMSSD between adjacent NN intervals
AVNN^c^	ms	Average value of NN intervals
SDNN^d^	ms	SD of NN intervals
pNN50^e^	—^f^	The proportion of number of pairs of successive NN intervals differing more than 50 ms divided by total number of NN intervals
LF^g^	ms^2^	Power of the LF band of the IBI^h^ signal (ie, 0.04-0.15 Hz)
HF^i^	ms^2^	Power of the HF band of the IBI signal (ie, 0.15-0.4 Hz)
LF:HF	—	Ratio of LF to HF

^a^NN: normal heart beat.

^b^RMSSD: root mean square of successive differences between normal heartbeats.

^c^AVNN: average of normal heartbeat intervals.

^d^SDNN: SD of normal beat-to-beat intervals.

^e^pNN50: percentage of successive beat-to-beat intervals that differ by more than 50 ms.

^f^Not available.

^g^LF: low frequency.

^h^IBI: interbeat interval.

^i^HF: high frequency.

#### Shimmer3 ECG

**Figure 1:**
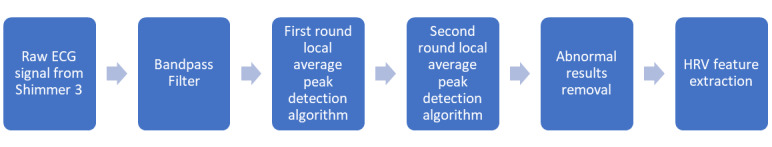
The electrocardiography analysis steps. ECG: electrocardiography; HRV: heart rate variability.

As previously mentioned, ECG was selected as the gold standard method to extract HR and HRV. In this regard, we chose Lead II (right arm–left leg) to extract the cardiac rhythm accurately. As the Oura Ring data were reported every 5 minutes, we also divided the ECG signals into 5-minute time windows. Then, we performed an ECG analysis to calculate the HR and HRV parameters for each window. Different steps of the analysis are illustrated in Figure 1.

The collected ECG signal quality is susceptible to artifacts generated by the user’s movements, poor electrode contact, or environmental noise. Such artifacts are inevitable in home-based monitoring, as users might engage in various physical activities. In this regard, we first used a Butterworth band-pass filter with 0.5-100 Hz cutoff frequencies to remove the artifacts that were not in the desired frequency range.

**Figure 2:**
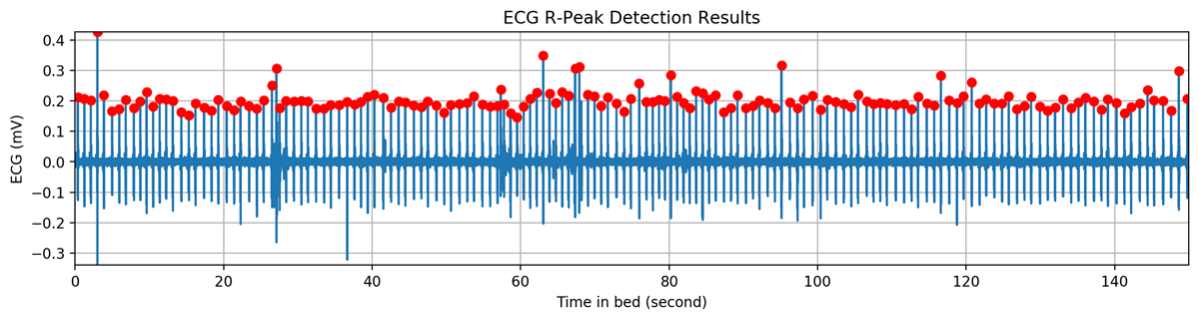
The peak detection result of a 5-minute time window. ECG: electrocardiography.

We designed a two-round peak detection method to extract the R peaks from the ECG signals. In the first round, the algorithm computes the average value of the ECG in a 5-minute window. It then detects all possible peaks, including the real R peaks and miscalculated peaks (made by P wave, T wave, or noises) using the average value as the threshold. In the second round, the algorithm calculates the average value of the peaks detected in the first round. Using the average value and the normal frequency (50-200 beats per minute) of heart beats, undetected R peaks were added and miscalculated peaks were removed. Our peak detection method obtains higher accuracy than the Pan–Tompkins [43] and Hamilton [44] algorithms. Figure 2 shows the peak detection results of a sample with a 5-minute ECG window.

**Figure 3:**
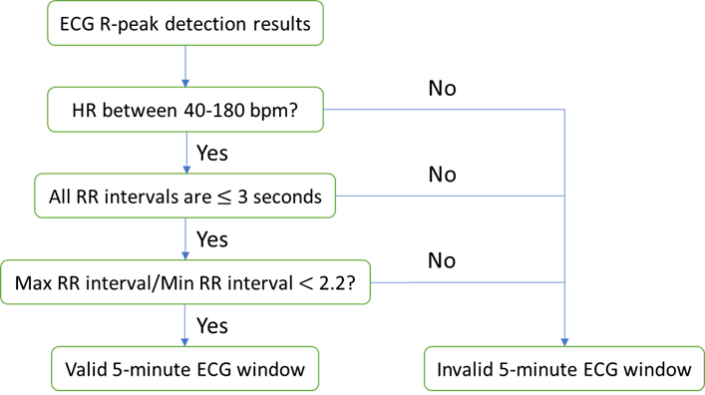
The pipeline of the electrocardiography validation method. ECG: electrocardiography; HR: heart rate; RR: respiratory rate.

However, our peak detection algorithm is inaccurate if the collected ECG includes too much noise (ie, low signal-to-noise ratio). To avoid such inaccurate peak detection, we developed a method to detect and remove the distorted signals and invalid peaks. The removal criteria are based on the normal range of the HR and RR intervals learned and modified from the ECG signal quality index [45]. Such a method is important in the analysis to prevent false peak detection and, subsequently, HR and HRV extraction. The method pipeline is illustrated in Figure 3.

#### Statistical Analysis

We used the Pearson correlation coefficient on pairwise HR and HRV parameters to investigate the linear relationship and comparability between the 2 devices. Moreover, a linear regression analysis was used to assess the accuracy of the HR and HRV parameters of the Oura Ring. We used Oura’s data points (HR and HRV parameters) to fit the linear regression line. Then, we computed the R-squared value (*r*^2^) using the regression line and corresponding baseline data points from the ECG to evaluate the closeness of the baseline data to the Oura Ring’s fitted regression line. Finally, the Bland–Altman analysis was used to illustrate and estimate the agreement between the 2 devices. This method provides mean bias, SD, and 95% CIs based on the differences between the ring and Shimmer3. We used Python and Python libraries, including Scipy [46], SKlearn [47], and Statsmodels [48] to program the statistical analysis functions.

### Research Ethics

The study was conducted according to the ethical principles of the Declaration of Helsinki and the Finnish Medical Research Act (No 488/1999). The study protocol was approved by the ethics committee (University of Turku, Ethics Committee for Human Sciences, Statement no: 44/2019). The participants were informed about the study, both orally and in writing, before written informed consent was obtained. Participation was voluntary, and all participants had the right to withdraw from the study at any time and without giving any reason. To compensate for the time used for the study, each participant received a gift card to the grocery store (€20; US $26.83) at the end of the monitoring period when returning the devices.

## Results

### Overview

The data of 35 participants (ie, 19 women and 16 men) were included in the analysis. In this study, an average 8.25 (SD 1.51) hours of nighttime sleep data were recorded for each participant to validate HR and HRV parameters. In the following, we first evaluated the HR and HRV parameters obtained from 5-minute segments. We then compared the average parameters during nighttime sleep.

### Comparisons of HR and HRV Parameters of Ring and Shimmer3 in 5-Minute Time Windows

We first investigated the correlation between HR and HRV parameters of the Oura Ring and Shimmer3 in 5-minute windows. The Pearson correlation coefficient and corresponding *P* values for the HR and HRV parameters are shown in Table 2. The HR and RMSSD between the Oura Ring and ECG were significantly correlated at *P*<.001. There were high positive relationships in the AVNN and pNN50 values, moderate positive relationships in the SDNN and HF values, and a low positive relationship in the LF and LF:HF ratio.

**Figure 4:**
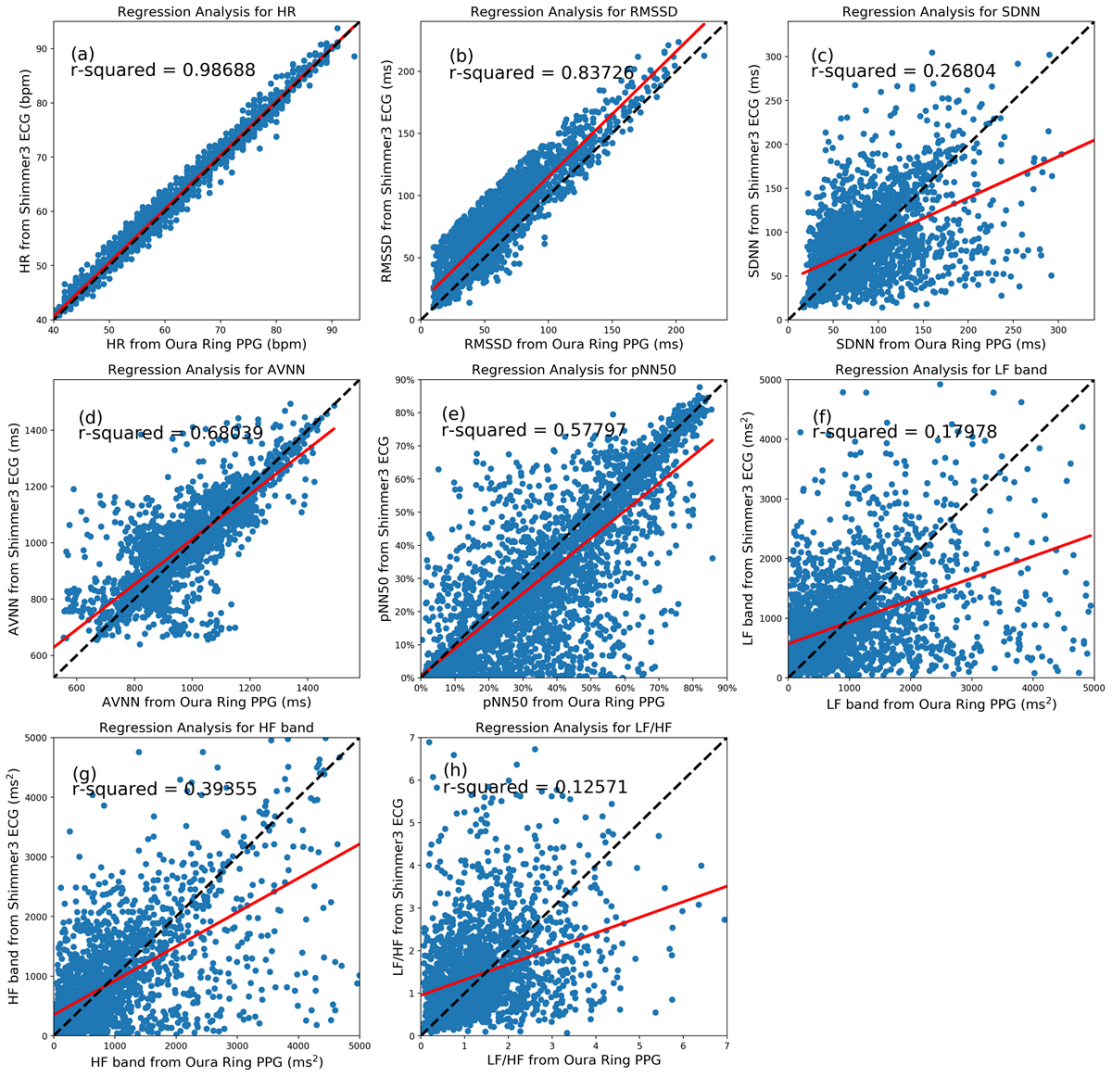
The scatter plots and regression analysis of the nocturnal heart rate and heart rate variability parameters collected from the Oura Ring and Shimmer electrocardiography in 5-minute segments. The regression lines and ideal lines are indicated in red and black, respectively. AVNN: average of all normal-to-normal intervals; ECG: electrocardiography; HF: high frequency; HR: heart rate; LF: low frequency; pNN50: percentage of successive beat-to-beat intervals that differ by more than 50 ms; PPG: photoplethysmography; RMSSD: root mean square of successive differences; SDNN: SD of beat-to-beat intervals.

We used regression analysis to examine the accuracy of the Oura Ring data compared with the ECG. The regression lines (in red) for 5-minute samples of all participants are illustrated in Figure 4. We also showed y=x lines (in black), indicating the best scenario where the values obtained from the Oura and ECG are equal. Moreover, *r^2^* values were reported, showing the scatter of the data around the regression lines. In this analysis, the fitted lines of the HR, RMSSD, AVNN, and pNN50 were close to the ideal lines, and their *r^2^* values were high. However, the data points of the SDNN, LF, HF, and LF:HF ratio are dispersed, and their *r^2^* values are relatively low.

**Figure 5:**
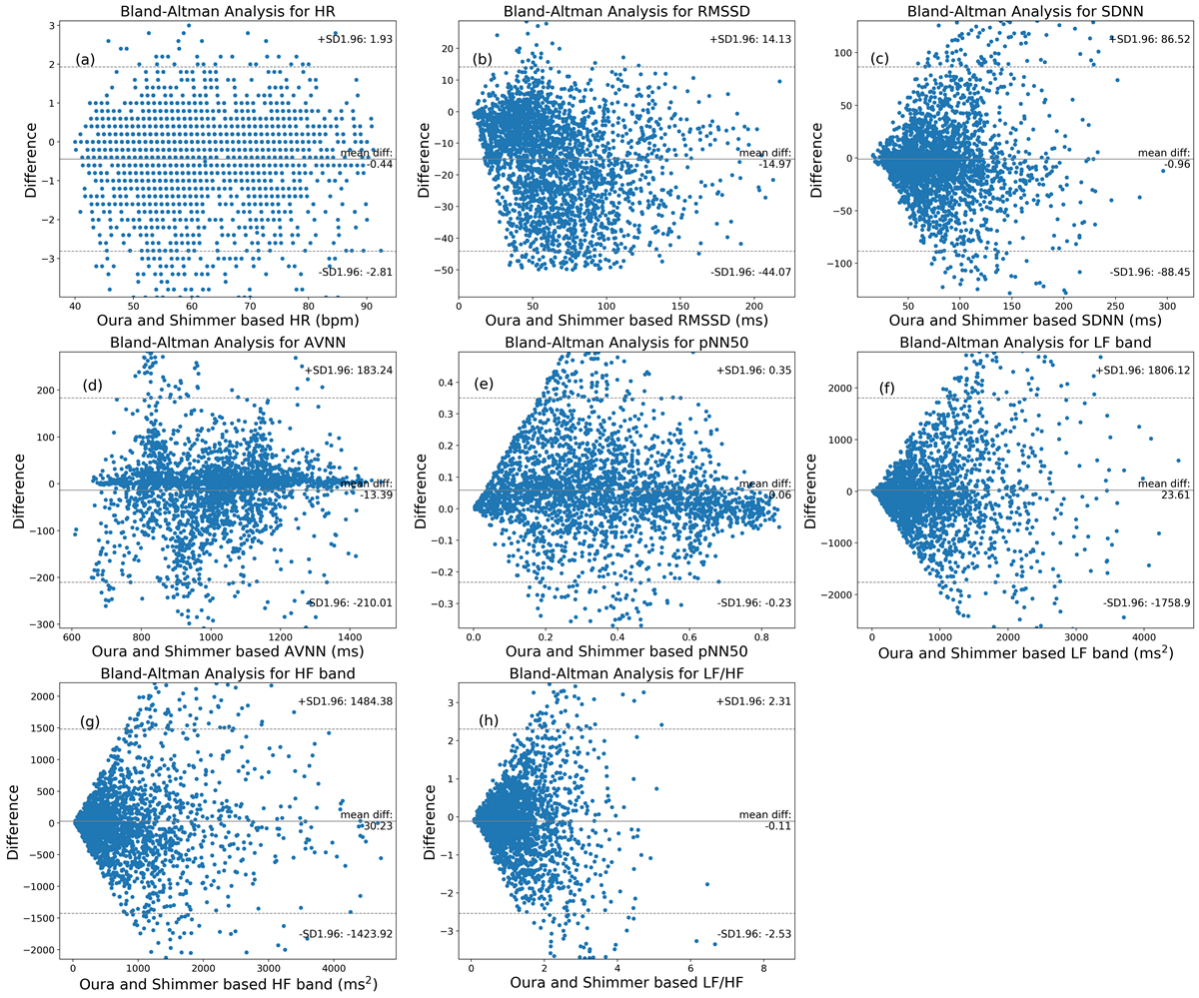
The Bland–Altman plots of the nocturnal heart rate and heart rate variability parameters in 5-minute segments obtained by the Oura Ring and Shimmer electrocardiography. AVNN: average of all normal-to-normal intervals; HF: high frequency; HR: heart rate; LF: low frequency; pNN50: percentage of successive beat-to-beat intervals that differ by more than 50 ms; PPG: photoplethysmography; RMSSD: root mean square of successive differences; SDNN: SD of beat-to-beat intervals.

In addition, Bland–Altman analysis was performed to investigate the agreement between the HR and HRV parameters extracted from the ring and ECG. Figure 5 shows the Bland–Altman plots. The mean bias and 95% CI are shown in Figure 5 and Table 2. The ring (on average) overestimated pNN50, LF, and HF values but underestimated the other parameters. The HR, RMSSD, AVNN, and pNN50 had narrow 95% CIs; however, SDNN, LF, HF, and LF:HF ratio had relatively wider 95% CIs.

**Figure 6:**
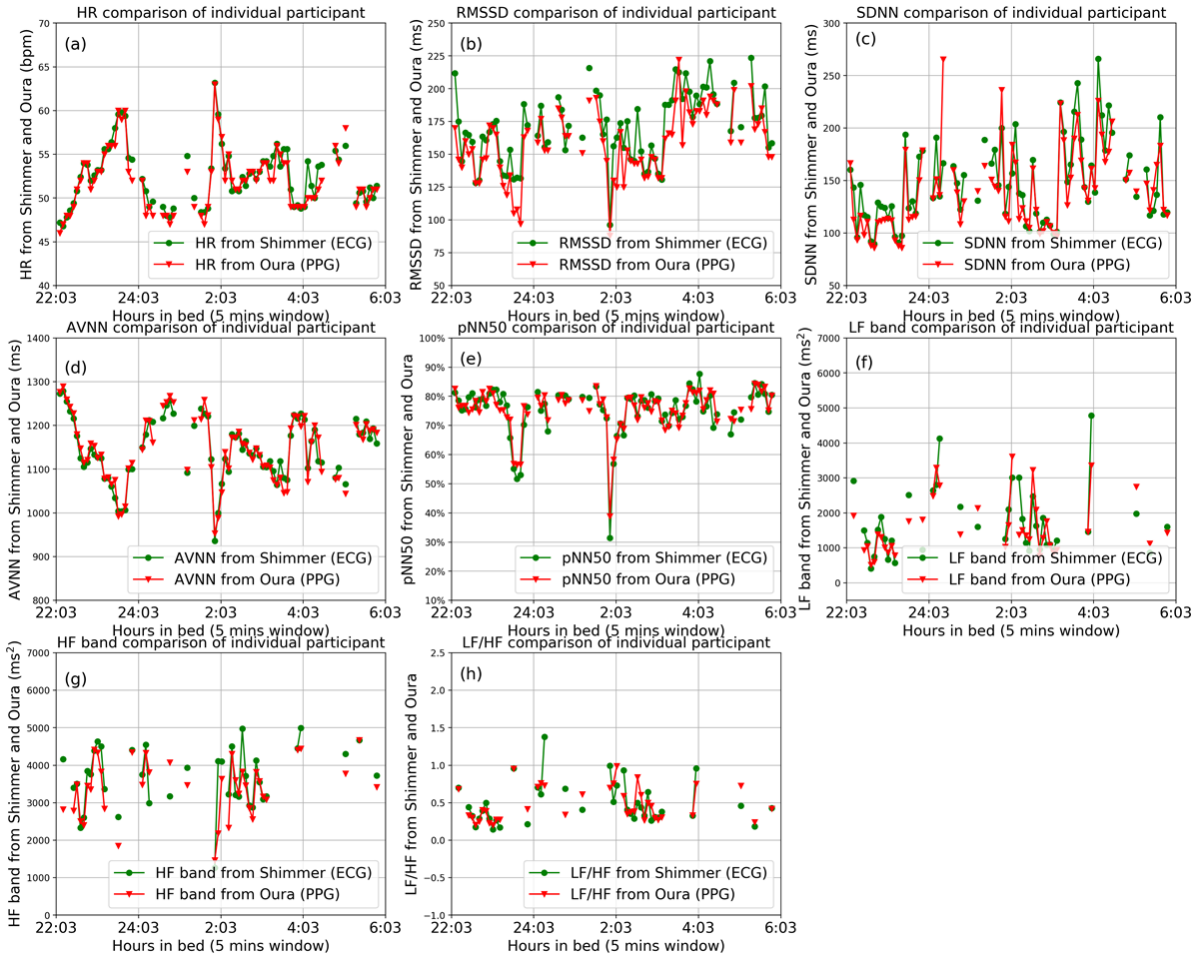
The nocturnal heart rate and heart rate variability parameters of one participant during a night sleep event. The data are extracted from the Oura Ring (in red) and Shimmer electrocardiography (in green). AVNN: average of all normal-to-normal intervals; ECG: electrocardiography; HF: high frequency; HR: heart rate; LF: low frequency; pNN50: percentage of successive beat-to-beat intervals that differ by more than 50 ms; PPG: photoplethysmography; RMSSD: root mean square of successive differences; SDNN: SD of beat-to-beat intervals.

We also demonstrated the nocturnal HR and HRV parameters of one participant (randomly selected) in Figure 6. The parameters were obtained from the 5-minute segments. Figure 6 shows how the collected parameters from the Oura Ring (in red) and from the ECG (in green) vary throughout the night. As indicated, there are missing values, particularly in the frequency domain parameters, because of the removal of low-quality segments of the ECG or IBI signals.

**Table 2:** Pearson correlation coefficient, *P* values, 95% CI, and mean bias for heart rate (HR) and HR variability parameters between Ring and Shimmer3 in 5-minute window time.

Parameters	Pearson correlation coefficient	*P* value	95% CI	Mean bias
HR	0.99341	<.001	−2.81 to 1.93	−0.44
RMSSD^a^	0.91502	<.001	−44.07 to 14.13	−14.97 ms
SDNN^b^	0.51772	<.001	−88.45 to 86.52	−0.96 ms
AVNN^c^	0.82486	<.001	−210.01 to 183.24	−13.39 ms
pNN50^d^	0.76024	<.001	−0.23 to 0.35	0.06
LF^e^ band	0.42401	<.001	−1758.9 to 1806.12	23.61 ms^2^
HF^f^ band	0.62734	<.001	−1423.92 to 1484.38	30.23 ms^2^
LF:HF ratio	0.35455	<.001	−2.53 to 2.31	−0.11

^a^RMSSD: root mean square of successive differences between normal heartbeats.

^b^SDNN: SD of normal beat-to-beat intervals.

^c^AVNN: average of normal heartbeat intervals.

^d^pNN50: percentage of successive beat-to-beat intervals that differ by more than 50 ms.

^e^LF: low frequency.

^f^HF: high frequency.

### Comparisons of Average HR and HRV Parameters of Ring and Shimmer3 During Sleep Time

We compared the average HR and HRV parameters per night sleep for the 2 devices to evaluate the overall errors. In this regard, we first extracted the correlation between the average HR and HRV parameters using the Pearson correlation test. Table 3 shows the Pearson correlation coefficients and corresponding *P* values. The correlation values of HR and RMSSD were very close to 1. Therefore, there were very strong positive correlations between the 2 devices. The AVNN, SDNN, pNN50, LF, and HF were higher than 0.8, showing high positive correlations between the 2 devices. There was also a moderately positive relationship in the LF:HF ratio.

**Figure 7:**
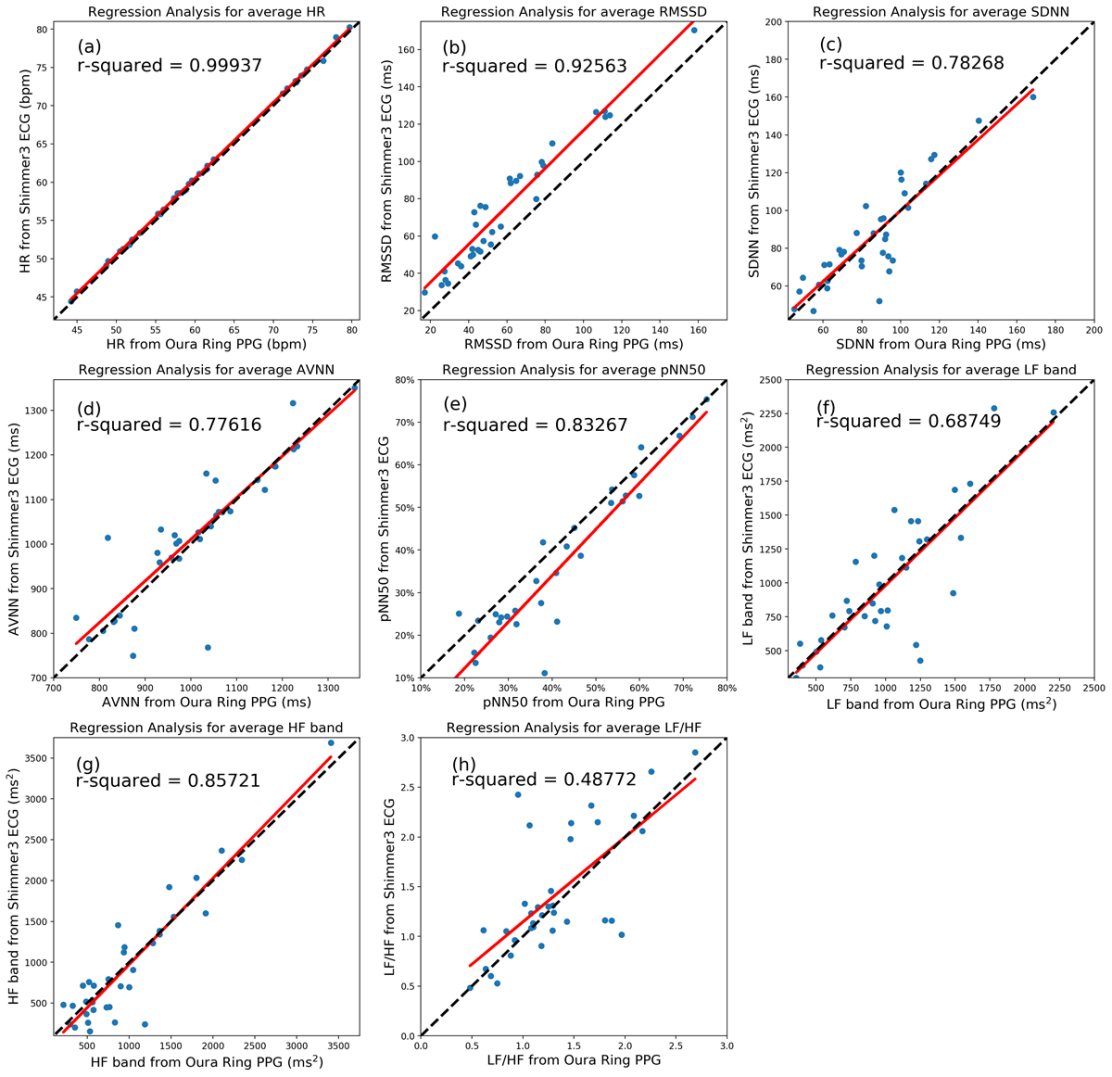
The scatter plots and regression analysis of the average heart rate and heart rate variability parameters (which are collected from the Oura Ring and Shimmer electrocardiography) per night sleep time. The regression lines and ideal lines are indicated in red and black, respectively. AVNN: average of all normal-to-normal intervals; ECG: electrocardiography; HF: high frequency; HR: heart rate; LF: low frequency; pNN50: percentage of successive beat-to-beat intervals that differ by more than 50 ms; PPG: photoplethysmography; RMSSD: root mean square of successive differences; SDNN: SD of beat-to-beat intervals.

We also used regression analysis to evaluate the average HR and HRV parameters throughout the night sleep period. Figure 7 illustrates the HR and HRV samples per night, the regression lines in red, the *r^2^* values, and the ideal lines (ie, y=x) in black. The *r^2^* values of the HR and RMSSD were greater than 0.9, indicating that the samples were near the regression lines. The *r^2^* values of SDNN, AVNN, pNN50, LF, and HF represent good fits. However, the *r^2^* value for the LF:HF ratio was 0.49.

**Figure 8:**
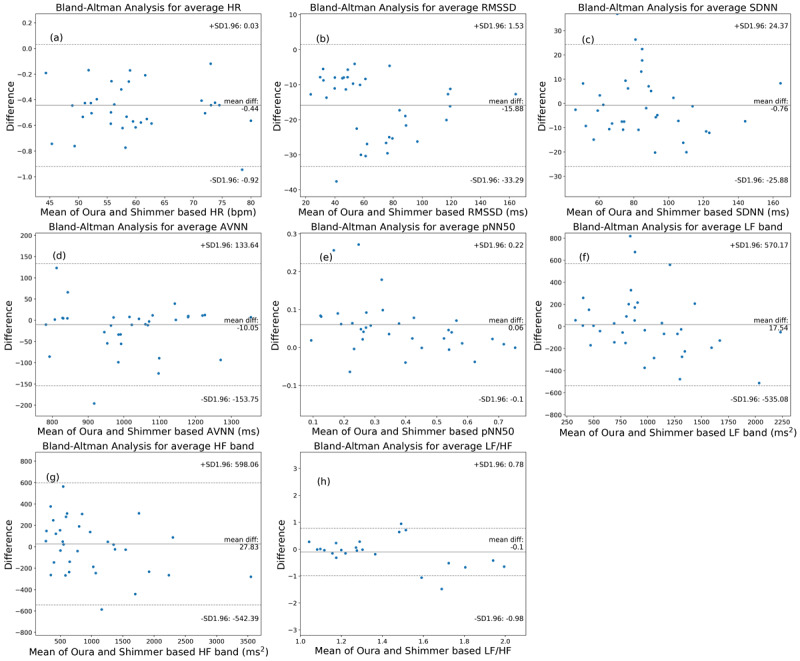
The Bland–Altman plots of the average heart rate and heart rate variability parameters (which are obtained by the Oura Ring and Shimmer electrocardiography) per night sleep time. AVNN: average of all normal-to-normal intervals; HF: high frequency; HR: heart rate; LF: low frequency; pNN50: percentage of successive beat-to-beat intervals that differ by more than 50 ms; PPG: photoplethysmography; RMSSD: root mean square of successive differences; SDNN: SD of beat-to-beat intervals.

In addition, we used the Bland–Altman analysis to investigate the differences between the average parameters per night sleep from these 2 devices (Figure 8). Table 3 shows the mean bias and 95% CI values. The results show that, on average, the ring overestimates pNN50, LF, and HF but underestimates the other parameters. Moreover, the 95% CIs of the HR, RMSSD, AVNN, and pNN50 were narrow, whereas the values were relatively wider for the SDNN and frequency domain parameters. These results are in accordance with the results presented in the previous section—Comparisons of HR and HRV Parameters of Ring and Shimmer3 in 5-Minute Time Windows.

**Table 3:** Pearson correlation coefficient, *P* values, 95% CI, and mean bias for the average heart rate (HR) and HR variability parameters per night collected from the Oura Ring and Shimmer3.

Parameter	Pearson correlation coefficient	*P* value	95% CI	Mean bias
HR	0.99968	<.001	−0.92 to 0.03	−0.44
RMSSD^a^	0.96210	<.001	−33.29 to 1.53	−15.88 ms
SDNN^b^	0.88469	<.001	−25.88 to 24.37	−0.76 ms
AVNN^c^	0.88010	<.001	−153.75 to 133.64	−10.05 ms
pNN50^d^	0.91251-	<.001	−0.1 to 0.22	0.06
LF^e^ band	0.82916	<.001	−535.08 to 570.17	17.54 ms^2^
HF^f^ band	0.92585	<.001	−542.39 to 598.06	27.83 ms^2^
LF:HF ratio	0.69837	<.001	−0.98 to 0.78	−0.1

^a^RMSSD: root mean square of successive differences between normal heartbeats.

^b^SDNN: SD of normal beat-to-beat intervals.

^c^AVNN: average of normal heartbeat intervals.

^d^pNN50: percentage of successive beat-to-beat intervals that differ by more than 50 ms.

^e^LF: low frequency.

^f^HF: high frequency.

## Discussion

### Principal Findings

In our analysis, we first validated the parameters extracted from the 5-minute PPG segments. 5-minute HRV recording, also known as short-term HRV analysis, is a measurement standard for extracting HRV parameters, such as RMSSD, SDNN, LF, and HF [4]. The LF:HF ratio is conventionally calculated via the 24-hour HRV recording [5]; however, it can also be collected in 5-minute recordings [4]. Our findings show relatively low mean biases for the HR and HRV parameters, where the Oura Ring overestimated pNN50, LF, and HF values but underestimated the other parameters. HR, RMSSD, AVNN, and pNN50 of the Oura Ring showed high positive correlations with the baseline, SDNN and HF showed moderate positive correlations, and LF and LF:HF ratio had low positive correlations.

However, the error variances of the parameters were different. The parameters provided by the Oura Ring dashboard (ie, HR and RMSSD) showed a relatively lower error variance compared with the HRV parameters extracted from the IBI signals. The error of HR is lower than that of RMSSD, which is in accordance with other studies showing that RMSSD is more sensitive to motion artifacts [35,49]. Among the parameters extracted from the IBI signals, AVNN and pNN50 showed moderate error rates compared with the baseline. However, SDNN, LF, HF, and LF:HF ratio had relatively higher error rates. The findings of the frequency domain parameters follow those of other studies, which show that these parameters are more sensitive to noise [30].

We also compared the average HR and HRV parameters during nighttime sleep. This comparison evaluates the long-term trends of HRV parameters and shows the validity of the parameters in a per-night analysis [34]. The mean biases per night were low, which is in accordance with the 5-minute recording analysis. In contrast, the error variances of the average values per night were considerably lower. This can be explained by the variance decrease because of averaging of the independent measurements. HR, RMSSD, AVNN, pNN50, SDNN, LF, and HF indicated high positive correlations, and the LF:HF ratio had a moderate positive correlation. To summarize, the average HR and HRV parameters per night were relatively more accurate than the parameters extracted from 5-minute segments. Our results showed that the Oura Ring could accurately measure HR and RMSSD in both the 5-minute and average-per-night tests. The ring provided acceptable nocturnal AVNN, pNN50, HF, and SDNN accuracy in the average-per-night test but not in the 5-minute test. In contrast, the LF and LF:HF ratio of the ring had high error rates in both tests.

### Comparison With Previous Studies

To the best of our knowledge, this is the first study to evaluate different HRV parameters of the Oura Ring in comparison with a standard ECG device. Kinnunen et al [34,35] focused on assessing the HR and RMSSD of the Oura Ring. In the 5-minute segment analysis, the HR and RMSSD were highly accurate. We obtained a higher *r^2^* for HR and a lower *r^2^* for RMSSD. Moreover, for the average-per-night analysis, we obtained almost the same *r^2^* for HR but lower *r*^2^ for RMSSD. Our results indicate a narrower 95% CI and a smaller mean bias difference for average HR, and a wider 95% CI and a greater mean bias difference for average RMSSD.

### Limitations

This study is limited to the nocturnal HR and HRV parameters, as the Oura Ring only provides the HR, RMSSD, and IBI values during sleep [34]. Future work should include the assessment of HR and HRV parameters during awake time. The PPG signals, and subsequently the parameters, might be distorted because of artifacts when the users engage in various activities and environments [50]. Such an evaluation is essential when using the ring in remote health monitoring and wellness tracking apps.

A total of 46 individuals participated in this home-based study, and data from 35 individuals were included in the analysis. However, this study was restricted to overnight data collection. Our future work will consider assessing the ring over the data collected over several days or weeks. This validation will provide a higher confidence level for the validity of the reported HR and HRV parameters.

Another limitation is the lack of generalizability of the results to nonhealthy individuals, as the study only included healthy participants. Recent studies have shown that the validity of wearable devices may be different for different population groups [51,52]. For example, atrial fibrillation affects the heart rhythms (irregular beats) of PPG [3]. Therefore, both time and frequency domain HRV parameters of individuals with atrial fibrillation are not the same as those of healthy people [36]. Consequently, the accuracy of the PPG-based atrial fibrillation methods should be investigated separately. Future directions for this study should include evaluating the PPG-based parameters acquired from individuals of different ages and with various health conditions.

### Conclusions

In this study, we comprehensively evaluated the validity of the HR and HRV parameters collected by the Oura Ring. Our results showed low mean biases for the 8 parameters. In the 5-minute test, the error variances of the parameters were different. The parameters provided by the Oura Ring dashboard (ie, HR and RMSSD) showed relatively low error variance compared with the HRV parameters extracted from the IBI signals. HR, RMSSD, AVNN, and pNN50 of the ring indicated high positive correlations with the baseline values; SDNN and HF had moderate positive correlations; and LF and LF:HF ratio showed low positive correlations. In contrast, the average-per-night test indicated considerably lower error variances than the 5-minute test for all parameters. The Oura Ring was capable of accurately measuring HR and RMSSD in both the 5-minute and average-per-night tests. The ring indicated acceptable nocturnal AVNN, pNN50, HF, and SDNN accuracy in the average-per-night test but not in the 5-minute test. In contrast, the LF and LF:HF ratio of the ring had high error rates in both tests. Future work should include assessing the HR and HRV of the ring in long-term monitoring of population groups with different health conditions.

## Abbreviations

AVNN: average of all normal heart beat intervals
ECG: electrocardiography
HF: high frequency
HR: heart rate
HRV: heart rate variability
IBI: interbeat interval
LF: low frequency
pNN50: percentage of successive beat-to-beat intervals that differ by more than 50 ms
PPG: photoplethysmography
RMSSD: root mean square of successive differences
SDNN: SD of beat-to-beat intervals
